# The Mechanical Properties Prediction of Poly [(3-hydroxybutyrate)-co-(3-hydroxyvalerate)] (PHBV) Biocomposites on a Chosen Example

**DOI:** 10.3390/ma15217531

**Published:** 2022-10-27

**Authors:** Grzegorz Janowski, Wiesław Frącz, Łukasz Bąk

**Affiliations:** Department of Materials Forming and Processing, Rzeszow University of Technology, Powstancow Warszawy 8, 35-959 Rzeszow, Poland

**Keywords:** PHBV, homogenization methods, PHBV properties, PHBV-hemp fiber biocomposites, polymer composites

## Abstract

This paper aims to experimentally determine the properties of the poly [(3-hydroxybutyrate)-co-(3-hydroxyvalerate)]—(PHBV)—30% hemp fiber biocomposite, which is important in terms of numerical simulations of product manufacturing, and to evaluate the mechanical properties by means of micromechanical modeling. The biocomposite was manufactured using a single-screw extruder. Specimens for testing were produced by applying the injection molding technology. Utilizing the simulation results of the plastic flow, carried out by the Moldflow Insight 2016 commercial software and the results of experimental tests, the forecasts of selected composite mechanical properties were performed by means of both numerical and analytical homogenization methods. For this purpose, the Digimat software was applied. The necessary experimental data to perform the calculations for the polymer matrix, fibers, and the biocomposite were obtained by rheological and thermal studies as well as elementary mechanical tests. In the paper, the method of determining selected properties of the biocomposite and the method of forecasting its other properties are discussed. It shows the dependence of the predicted, selected properties of the biocomposite on the filler geometry assumed in the calculations and the homogenization method adopted for the calculations. The results of the work allow for the prediction of properties of the PHBV biocomposites—hemp fiber for any amount of filler used. Moreover, the results allow for the estimation of the usefulness of homogenization methods for the prediction of properties of the PHBV-hemp fiber biocomposites. Furthermore, it was found that for the developed and tested biocomposites, the most effective possibility of mechanical properties prediction is using the Mori-Tanaka homogenization model, which unfortunately has some limitations.

## 1. Introduction

One feature of the composites is that their micro-scale properties significantly affect the macroscopic properties [1,2,3,4]. An ability to describe microstructural phenomena results in a better understanding of the macroscopic behavior of the material. However, it should be noted that the microstructural properties of new materials are often not precisely known, thus it is necessary to make certain assumptions, including the selection of appropriate micromechanical models. The use of micromechanical models makes it possible to successfully predict the mechanical properties of composites taking into account the orientation of fillers with their variable content and with the use of various homogenization methods. Moreover, mechanical properties of foamed composites filled with continuous fibers as mats and fabrics are predicted. The possibility of making these types of calculations is provided by Digimat software, among others, which is the calculation algorithm using models of both analytical and numerical homogenization [5].

The second direction of the application of micromechanical models may be forecasting of polymer composites with short fibers processing using Autodesk Moldflow Insight (AMI) software. The current capabilities of simulation software allow, in some areas, for the verification of experimental data [6,7] to assess the effectiveness of the application of appropriate micromechanical models, as well as to assess the possibility of manufacturing a specific product. The injection molding simulation of products made of composites filled by short cellulose fibers is a problematic issue. This analysis requires the introduction of many input data, such as: Rheological data, pVT characteristic, thermal properties of the composite, etc. In addition, it is required to enter detailed data related to the geometric properties of the fibers to perform calculations related to their orientation in the polymer matrix. The fibers orientation in the polymer matrix is one of the main factors that condition the mechanical and processing properties of composites. The knowledge of the fibers orientation allows one to determine the proper dimensions of the forming cavities, as well as to design, with the current possibilities of CAE software, some physical and mechanical properties of composite products [8,9,10,11]. The proper use of micromechanical models allows for the assessment of fibers flow during processing and their orientation after processing. The Folgar-Tucker model is used as the standard in calculating the fiber orientation. However, recent scientific works indicate that this model overestimates the values of the orientation tensor components in concentrated suspensions, which are polymers in this model [12,13,14]. The reduced strain closure (RSC) model is a modified approach to the indicated problem and is increasingly widely used in CAE software. In turn, the elastic properties of the composite in CAE software, dedicated to analyze the injection molding process, are calculated on the basis of the Halpin-Tsai micromechanical model [15,16], and the Rosen-Hashin model is used to determine the thermal expansion coefficients [17].

Primitively, the possibilities of properties that describe composite materials are focused only on carrying out appropriate experiments for the known material. Then, the possibility of using analytical methods were undertaken. They were characterized by great limitations. Moreover, often with the use of these models, the results consistent with the experiment were not recorded. In the current trend, the possibility of using CAE techniques for properties predicted in the microstructural aspect, including homogenization methods is observed. The element of the tested material subject to averaging, i.e., homogenization is defined as a representative volume element (RVE). The RVE must meet the main condition, i.e., contain a matrix and an inclusion, to accurately reflect the properties of the material being tested. The possibility of predicting the properties of materials using homogenization methods may contribute to a significant reduction in the number of time-consuming and costly experiments and may contribute to the improvement of the developing possibilities and designing new materials [18,19,20]. Research on composite properties based on homogenization methods was carried out on the basis of a number of assumptions regarding internal phenomena in the material microstructure. In the descriptions of Rayleigh and Maxwell [21,22], the macroscopic properties of materials consisting of a spherical particle embedded in a matrix were defined. This assumption laid the foundation for the definition of elementary assumptions of the analytical homogenization models. One of the basic models of this type is the Voigt model [23], which assumes that the deformation field in the mass of a heterogeneous material sample is homogeneous, which results in an effective definition of the averaged properties of the analyzed materials. Based on Voight’s assumption, the Eshelby model was formulated, which was based on the concept of self-deformation. It was used to determine a solution to the problem of a single inclusion embedded in an infinite matrix of material subject to uniform external stress [24]. The Eshelby model was the basis for the development of many homogenization methods, which were based on the interaction calculation between the matrix and the inclusion with a specific geometry. The most popular homogenization model is the Mori-Tanaka model [25], whose assumption is based on the Eshelby’s approximate solution. In this model, the strain concentration tensor of all inclusions, with the average value of their deformation in relation to the average value of the matrix deformation, is equal to the strain concentration tensor for a single inclusion. The area of the tested material is interpreted as infinite. The average deformation of the matrix in the analyzed RVE can be interpreted as the deformation for the entire area of the matrix occurrence on the macroscopic scale. Based on this statement, Benveniste [26] advanced an interpretation of the Mori-Tanaka model. According to its assumption, each RVE inclusion is interpreted as an individual inclusion in the polymer matrix. The model was extended with additional assumptions to improve the predicted properties of materials analyzed in the nonlinear range, and it can be referred in this case as the second order model. For this correction, the following assumptions must be met: The matrix is characterized by a slight reinforcement, and the fiber is the main reinforcement. Moreover, there is a clear difference in stiffness between the matrix and the fibers. Failure to meet these conditions simultaneously results in the lack of significant differences between the results of the predicted material properties between the applied models of the 1st and 2nd order [27,28]. Furthermore, it is important that this model is highly effective in predicting the properties of two-phase composites with filler amounts up to 25%, and even higher values [29,30,31]. The “double inclusion” model was formulated by Nemat Nasser and Hori [32], where the main assumption of this model is the fact that each inclusion (I) (with C1 stiffness) is covered by a matrix (Io) (with the stiffness C0), and outside this system there is a reference center (with the stiffness Cr). For composites analyzed in the elastic range, this model usually provides a good prediction of properties, taking into account large inclusions in the matrix, aspect ratio, and differences in stiffness.

Due to the growing computing powers of PCs, the development of numerical methods can be noticed, which can be used to perform microstructural calculations based on homogenization methods [33] at the three-dimensional level of the analyzed material. This modeling is devoid of the fundamental limitations of flat area computing technology, but is significantly more demanding in terms of memory and computing power. One of the main types of finite elements used in microstructural calculations is the voxel element [34]. Each finite element is assigned to the analyzed area of the RVE material, where the composite properties are predicted with the use of appropriate homogenization models. It is difficult to indicate the most effective method of homogenization, which results from the fact that each composite material, in particular composites with cellulose fillers, are characterized by specific properties and high variability in properties. However, when defining the right input data and choosing the right homogenization method, there is a high probability of obtaining results that correlate well with the experiment.

New polymer materials with ever higher properties and lower production costs are still being sought. The growing ecological awareness prompts the search for environmentally friendly materials. This shapes the direction of production and improvement of a new type of plastic: From non-renewable, difficult to degrade or completely non-biodegradable to renewable polymers of natural origin and fully biodegradable. Biocomposites prepared with the use of a filler in the form of plant fibers and a biopolymer matrix can be one of the methods of reducing production costs and obtaining the appropriate properties while maintaining full biodegradation [35]. The current trend related to counteracting the problem of management of petrochemical plastic waste prompts the development of modern polymer materials [36,37,38,39,40], whose properties are initially unknown. An example of this can be the poly [(3-hydroxybutyrate)-co-(3-hydroxyvalerate)]—(PHBV)-hemp fiber (PHBV-hemp fiber) biocomposite, which is characterized by its natural origin and the possibility of full biodegradation [41,42,43]. Due to the fact that hemp fibers are natural ones, they are characterized by a lower repeatability and homogeneity of the structure, which results in a greater dispersion of properties. Another problematic issue is the possibility of predicting these types of composite properties by means of homogenization methods. One of the requirements is to determine many new data and evaluate many factors that define the effectiveness of the results which should be as compatible with the experiment as possible. The above problem was carried out by the authors of the paper and described in this publication.

## 2. Materials and Methods

### 2.1. Research Materials

PHBV, under the trade name Enmat Y1000 of Helian Polymers (Belfeld, The Netherlands) in the form of a powder, was used as the polymer matrix in the produced composites. The molar proportion of HV in the biopolymer was 8%, the density of the biopolymer was 1250 kg/m^3^, and the softening point ranged from 165 to 175 °C [44,45].

Hemp fibers with a mass fraction of 30% were used as a filler in the polymer matrix. Hemp fibers were provided by EKOTEX company (Kowalowice, Poland) and were 1 mm in length. The average ratio of the length to the diameter of the fibers (L/d) was approx. 10 (for L = 1 mm).

### 2.2. The Input Data Determination

Manufacturing of PHBV biocomposites filled with hemp fiber and the material data of hemp fiber are presented in the authors’ research work [41,42,43,46]. The work involved the production of a PHBV biocomposite with a mass fraction of 30% of hemp fiber.

Prior to biocomposite manufacturing, the fibers were surface modified with 10% sodium hydroxide solution to improve adhesion between the fibers and matrix. The alkalization was carried out for 1 h in a rotor device, where the fibers were mixed with the solution. Then, the fibers were washed with water until a pH value of 7 and filtered off using a centrifuge. Thereafter, they were dried at 90 °C and sieved. A single-screw ZAMAK EHP 25E extruder (produced by ZAMAK Mercator company, Skawina, Poland) was used for the production of biocomposites. The screw extruder was equipped with the five temperature-controlled zones, i.e., head = 175 °C, zone 3 = 170 °C, zone 2 = 160 °C, zone 1 = 150 °C, and feed zone = 35 °C. The process was carried out at a speed of 100 RPM. To determine the mechanical, thermal, and rheological properties, the samples in the dog bone shape, produced by the injection process with the Dr Boy 55E injection molding machine produced by BOY Maschines Inc. (Exton, PA, USA), were used. The processing parameters of injection molding were as follows: Mold temperature = 85 °C, melt temperature = 185 °C, cooling time = 25 s, packing time = 25 s, packing pressure = 30 MPa, and injection rate = 35 cm^3^/s. To determine the strength properties of biocomposites, the Zwick Z030 universal testing machine was used in accordance with the ISO 527-1 standard. The used materials were dried prior to the extrusion and injection molding process for 3 h at 90 °C.

The series of samples consisted of seven pieces in compliance with statistical analysis. Based on the test results, the Young’s modulus (E) and stress-strain characteristic were analyzed. The results were statistically analyzed and the following were determined: Arithmetic mean ($\bar{x}$) = 6992.31 MPa, standard deviation (s) = 199.44, and coefficient of variation (V) = 2.85.

#### 2.2.1. Viscosity Curve

**Figure 1:**
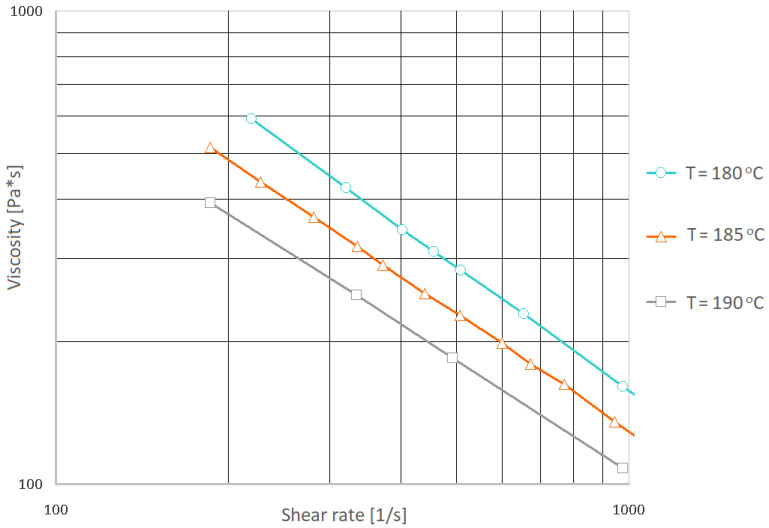
Viscosity curves for the K30 biocomposite determined in real conditions (in the injection mold).

The Ceast Smart Rheo 2000 capillary rheometer produced by Instron Inc. Europe (Buckinghamshire, UK) was used to determine the viscosity curve of the PHBV-hemp fiber biocomposite. The tests were performed at the temperatures of 180, 185, and 190 °C. Unfortunately, good results were not obtained due to excessive pressure pulsation during the measurement and the lack of stabilization of the material flow through the rheometer nozzle. Therefore, for this purpose, an injection mold equipped with temperature and pressure sensors integrated with the Priamus software to monitor the entire process was used. The research stand was presented in [47]. The tests were performed with a variable flow rate of the composite, i.e., from 10 to 70 cm^3^/s. Based on the obtained results, the viscosity curves were determined (Figure 1).

Moreover, based on the determined viscosity values, the coefficients of the seven-parameter Cross-WLF mathematical model were determined (Table 1).

#### 2.2.2. The pVT Characteristics

Based on the viscosity values, the coefficients of the seven-parameter Cross-WLF mathematical model were determined (Table 1). The thermodynamic pVT characteristic was determined by means of the Instron Ceast Smart Rheo 2000 capillary rheometer, for the following temperatures: 100, 120, 140, 150, 160, 170, 180, and 190 °C. The research stand was presented in [47]. Based on the obtained dependencies, the parameters of the Tait model were determined (Table 2).

#### 2.2.3. Thermal Properties

**Figure 2:**
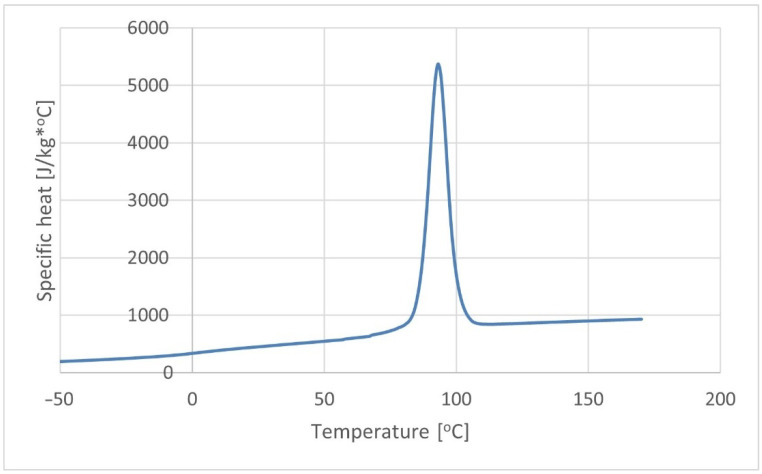
Specific heat of the K30 biocomposite.

**Figure 3:**
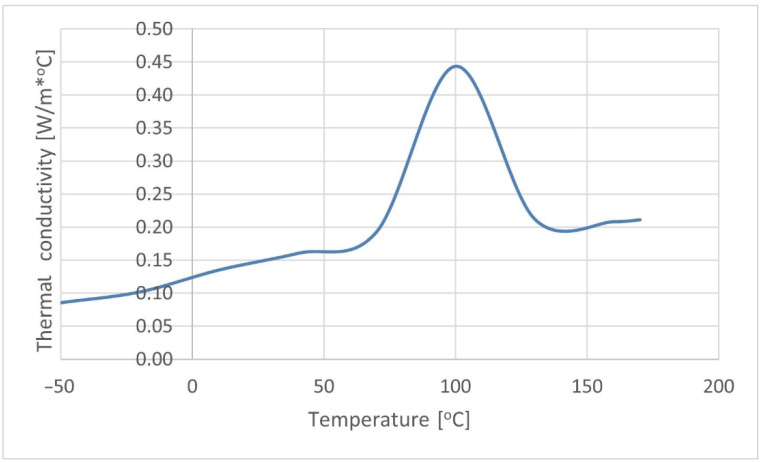
Thermal conductivity of the K30 biocomposite.

The composite thermal conductivity and specific heat were determined on the basis of differential scanning calorimetry (DSC), as shown in Figure 2 and Figure 3. The tests were carried out using scanning microcalorimeter the Q2000™ from TA Instruments, Inc. (New Castle, DE, USA). The research was conducted in accordance with the methodology presented in [48].

### 2.3. The Fiber Orientation Numerical Analysis

The Autodesk Moldflow Insight 2016 software was used to simulate the injection molding process. The fiber orientation analysis was performed based on the Folgar-Tucker model. Moreover, the elastic properties of the biocomposite were calculated on the basis of the Halpin-Tsai micromechanical model. Furthermore, the elastic properties of the polymer matrix and fibers, as well as fiber content and the aspect ratio were taken into account. The Rosen-Hashin model was chosen to determine the values of thermal expansion coefficients. Considering the dynamics of changes in fiber orientation, the reduced strain closure (RSC) model was used.

**Figure 4:**
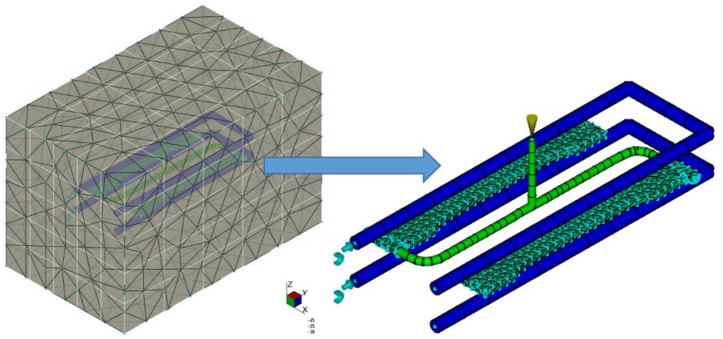
Numerical model of injection mold (FE of the tetra type).

The discretized 3D model of the injection mold is shown in Figure 4.

The injection molding processing parameters are presented in Table 3.

**Figure 5:**
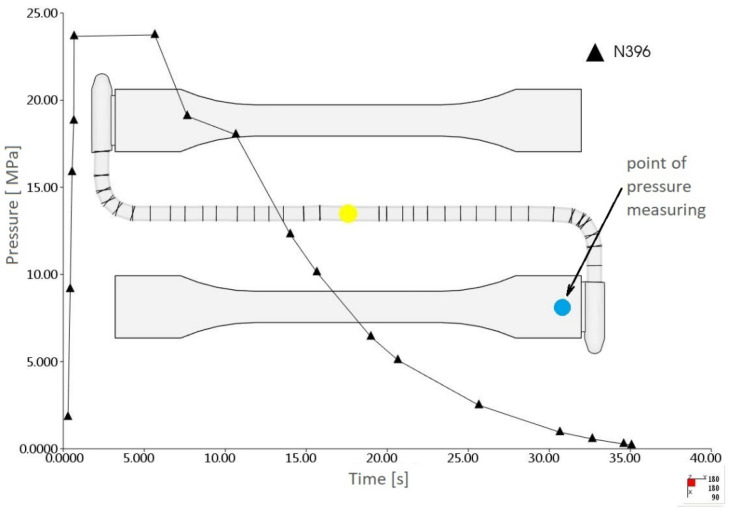
The pressure profile measured in the mesh FE node corresponding to the location of the pressure sensor.

**Figure 6:**
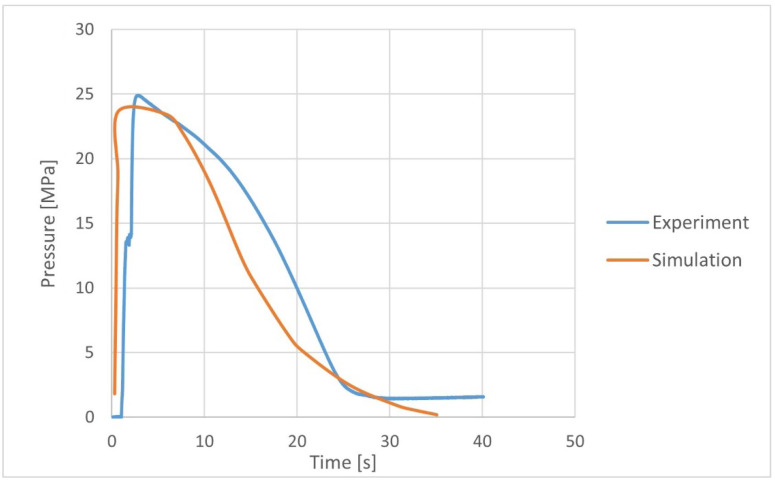
Comparison of cavity pressure profile from the experiment and numerical analyses.

Based on the polymer flow simulation, the pressure profile in the mold cavity was obtained, also in places where the pressure sensor was installed (Figure 5). Direct measurement of pressure in the mold cavity allowed for the verification of the numerical analysis results. A significant agreement was found between the pressure profile obtained in the mold cavity on the basis of the experiment and numerical analysis (Figure 6), which confirmed the correctness of the input data selection.

**Figure 7:**
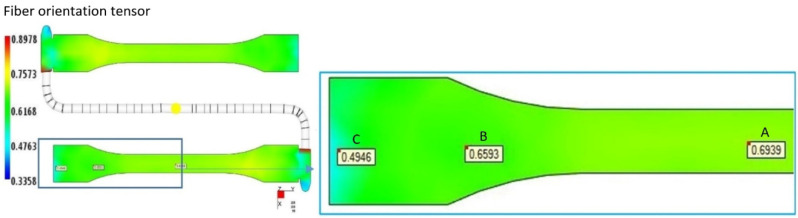
Tensor a_11_ value in selected zones of the mold cavity.

The fiber orientation was determined in selected zones of mold cavity. As a result of the calculations, the probability distribution of the fibers in the polymer matrix described by the orientation tensor was obtained (Figure 7). The values of the tensor components are interpreted as the probability of laying the fibers along and transversely to the flow direction of the biocomposite and on the thickness of the mold cavity. The fiber orientation tensor value equal to 1 determines the greatest probability of the orientation of fibers in a given direction. In the area (region A) of the tested sample, the value of the a_11_ orientation tensor is 0.694. With the change in the geometry of the cavity (region B), an increased degree of fiber disorder is noticeable. It is emphasized by a slight decrease in the value of the a11 tensor to 0.659. For the orientation of the fibers in region C, the value of the a_11_ orientation tensor is the lowest (0.495). The highest degree of fiber disorder in the polymer matrix occurs here, which results from the impact of the material stream against the most distant walls of the mold cavity, which takes place at the lowest pressure.

Verification of the pressure profile in individual areas of the mold cavity with the results of the experiment proves that the input data necessary to simulate the process are correctly determined. This allows for conducting numerical simulations to predict the course of the injection molding process of the PHBV-hemp biocomposite (30 wt%—the so-called K30) with other adjustable parameters, which limit the number of costly attempts to produce products from the biocomposite. In addition, the data obtained regarding the orientation of the fibers in the polymer matrix are used to conduct microstructural analyses that allow for the prediction of the properties of biocomposites with different percentages of filler.

## 3. The Micromechanical Modeling

The heterogeneity of composite materials is a problem, not only at the level of material processing and description of the structure, but also at the property prediction level, i.e., on the computational level. One of the solutions to this problem is micromechanical modeling, which provides the ability to predict interactions between the micro- and macro-scale of the analyzed material. The micromechanical calculations to predict the properties of the composites were performed using the Digimat software. In this way, the properties of the biocomposite with 30 wt% of hemp fiber (so-called K30) were determined and obtained using a single-screw extruder. These properties were verified by an experiment. Based on the verification with the results of experimental tests, the optimal method of homogenization was selected. This method was used to predict the properties of potential biocomposites, taking into account the variable filler content.

### 3.1. Data for Microstructural Analysis

To perform microstructural analysis, the matrix and fiber properties were defined. The experimental data from the uniaxial tensile test (Table 4) were introduced to the matrix description and the elastic-plastic model with isotropic symmetry was selected [49,50,51,52].

The properties of hemp fibers necessary for the numerical analysis of the injection molding process were determined on the basis of a literature review and our own research (Table 5) [53,54].

Density, Young’s modulus, and Poisson’s ratio for the filler and hemp fibers were taken into account. For hemp fibers, an elastic model with transversal isotropic symmetry was used. The value of the fiber orientation tensor and the L/d ratio for the fiber, which were determined experimentally, were introduced. The mass content of the hemp fibers in the polymer matrix was determined as 30%. The dimension of the representative volumetric element (RVE) and the number of finite elements of the Voxel type were defined (Table 6).

### 3.2. Results Interpretation

Micromechanical analyses were performed for four different types of fiber geometry: Ellipsoidal, cylinder, sphero-cylinder, and curve cylinder. Equal RVE dimensions are defined for all possible fiber shapes. A representative volume element was discretized for each of the four analyses using the same number of voxel finite elements. The visualization of RVE before and after discretization for four specific types of fiber geometry is shown in Table 7.

**Figure 8:**
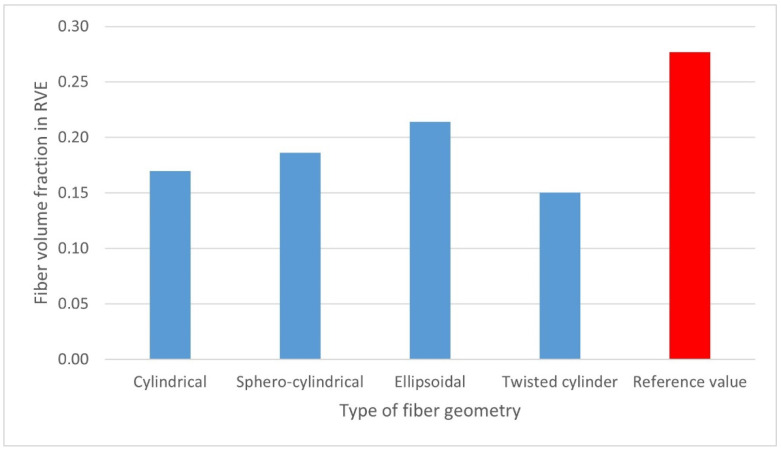
Calculated fiber volume content of the analyzed RVE in relation to the reference value of 27.7% for different types of fiber geometry.

One of the computational problems for the K30 composite was to ensure the correct distribution of the fibers in the RVE at a specific volume content of 27.7% (corresponding to 30% by weight). Calculations of the fiber content were made for each type of their geometry. On the basis of Figure 8, it was found that the choice of the fiber geometry significantly influenced the calculated volumetric content. This may be a consequence of difficulties in arranging fibers with the appropriate geometry in the RVE. Their largest volume share was 21.4%—for the ellipsoidal geometry, while the smallest volume share, i.e., 15%, was obtained for fibers with twisted cylinder geometry.

**Figure 9:**
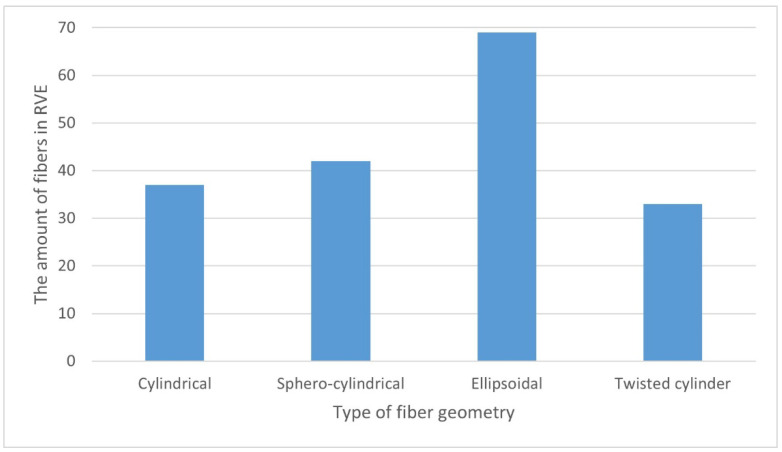
Dependence of the amount of fibers in the RVE on their geometry.

For a given mass/volume share, the actual number of fibers distributed in the RVE was analyzed (Figure 9). When analyzing the results, it can be seen that for the geometry of the twisted cylinder, only 33 fibers remained arranged in the RVE, which resulted in the lowest value of the volume fraction of fibers in the polymer matrix. On the other hand, the highest number of fibers in RVE was noted for fibers with an ellipsoidal geometry and 69 fibers which, in turn, translated into the highest obtained volume share.

One of the most important parameters that determine the micromechanical properties of composites with fibers is the fibers orientation. The values of the a_11_, a_22_, and a_33_ orientation tensors were obtained on the basis of the numerical simulation of biocomposite injection molding process. The highest compliance of this tensor value with the value obtained in Digimat FE was attained for established fibers with a sphero-cylindrical geometry (Table 8).

#### 3.2.1. The Fiber Geometry Influence

**Figure 10:**
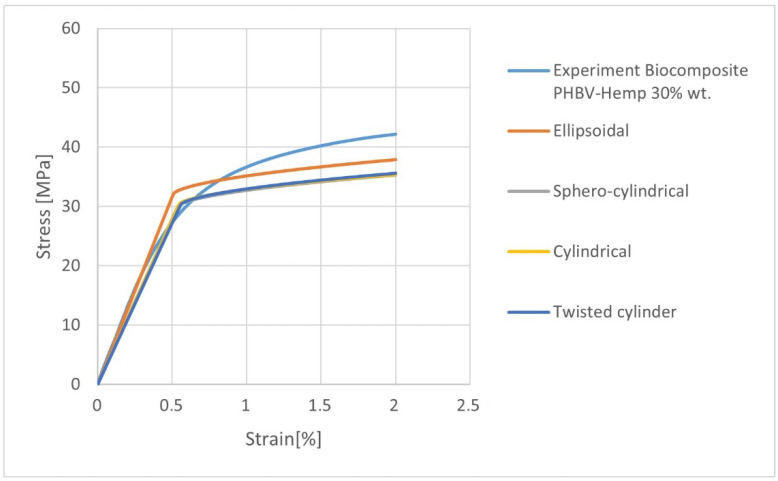
Stress-strain characteristics for K30 biocomposites with variable types of fiber geometry.

In the calculations, the influence of the fiber geometry on the mechanical properties was assessed (Table 9, Figure 10). High compliance with the experiment was found for the biocomposite filled with ellipsoidal fibers. The value of Young’s modulus in the longitudinal direction (E1) differs from the experimental value ($\bar{x}$ = 6992.31 MPa, s = 199.44, and V = 2.85) by approx. 6%. On the other hand, the lowest consistency of the results can be noticed for the biocomposite filled with fibers with the geometry of a twisted cylinder—the value of Young’s modulus E1 differs by approx. 28% from the real value. In the case of the calculated density, very high compliance of the results with the experiment was obtained, where the value from the experimental tests was 1289 kg/m^3^ (Table 9).

The selection of the appropriate fiber geometry in the RVE determines their effective volumetric content. Moreover, their geometry influences their amount in RVE. The large difference in their number obtained as a result of the calculations (depending on the adopted geometry) is the result of the “fiber distribution” algorithm used in Digimat in RVE. This algorithm aims to obtain the most consistent fiber volume content in RVE with the assumed value. Therefore, at this stage of calculations, the volume dimensions of a single fiber and the dimensions of the RVE are important.

The calculated values of the orientation tensor after transformation from Moldflow to RVE model in Digimat FE are slightly different. The best compatibility of the results in the case of numerical homogenization (Digimat FE) is provided by the choice of fibers with an ellipsoidal geometry. This fact may result from the largest volume fraction (closest to the real value) of the filler and the amount of fibers in the polymer matrix, based on these assumptions which were estimated by the software.

#### 3.2.2. Homogenization Method Influence

Analytical methods of homogenization (Mori-Tanaka 1st order, 2nd order, and double inclusion) were used in the research to predict the mechanical properties of the biocomposite. The predicted properties (Table 10) were compared with the results obtained by the numerical homogenization method on the example of the ellipsoidal geometry of the fibers. The mechanical properties and the stress-strain characteristics were compared. It was noticed that in the elastic range, the choice of the 1st and 2nd order Mori-Tanaka homogenization model allowed for the attainment of very convergent results. Moreover, the obtained data were compared with the experiment on the basis of Young’s modulus in the longitudinal direction E1. They only differ by 3.5% from the experimental value ($\bar{x}$ = 6992.31 MPa, s = 199.44, and V = 2.85). A very high result agreement of composite density prediction, compared with the experimental data (1289 kg/m^3^), was also obtained. The density value calculated using the Mori-Tanaka models (1st and 2nd order) differed only by 0.078% from the value obtained from the experiment.

**Figure 11:**
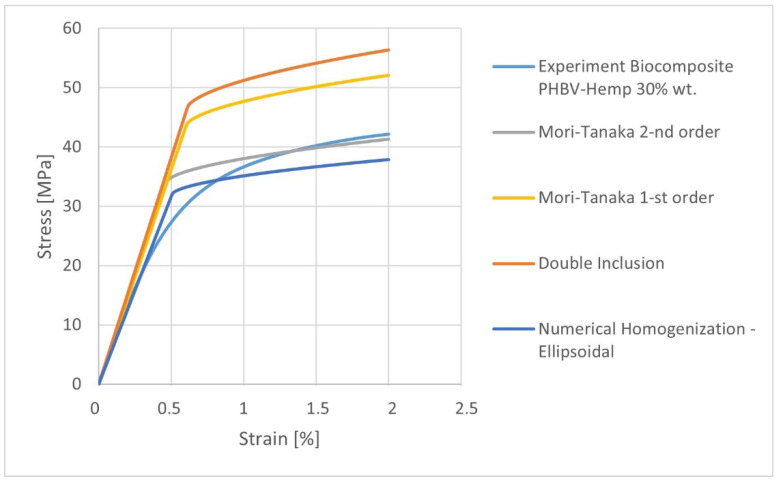
The stress-strain characteristics for K30 biocomposites calculated using analytical and numerical homogenization methods.

Taking into account the predicted stress-strain characteristics (Figure 11), the best agreement with the experiment for the 2nd order Mori-Tanaka model can be observed.

The results of the predicted properties of biocomposites indicate that the properties calculated by the 1st and 2nd order Mori-Tanaka homogenization models coincide in the elastic range. In the case of stress-strain characteristics, the results are different in the non-linear range. Moreover, it should be noted that the choice of the 2nd order Mori-Tanaka homogenization model enables the attainment of the results which are most consistent with the experiment, as compared to the other analytical and numerical homogenization methods.

## 4. Discussion

It is problematic to predict the mechanical properties of hemp fiber composites. This difficulty mainly results from the lack of skills and experience in selecting the appropriate micromechanical models. Averaging methods are used to determine the mechanical properties of composites. These are analytical and numerical homogenization models that are also used by the Digimat software. Using the above-mentioned methods for a small area of material containing fiber and matrix, their properties are averaged which, in turn, are transferred to the macro-scale to predict the properties of the entire material. This allows for the reduction in costly experimental studies. It should be mentioned that the most popular homogenization methods are characterized by a different degree of prediction of the actual properties of materials.

Furthermore, a significant problem is the effectiveness of the applied homogenization methods in relation to the filler content in the matrix. This is especially important for numerical methods, where each inclusion is separately modeled in RVE. It generates a very large finite element mesh and can be a problem even for high-performance PCs. On the other hand, in the case of analytical methods, it is known from the literature that, for example, the Mori-Tanaka model provides high efficiency of the results up to the mass content of the filler amounting to a maximum of approx. 25% [29,30,31]. Therefore, it is interesting to note how the variable content of the filler affects the effectiveness of predicting the mechanical properties of polymer composites.

The work of Das Lala [55] has developed a composite reinforcing biodegradable rubber seed shell, cashew nutshell, and walnut shell powder in epoxy resin. The filler content was 5, 10, 15, 20, and 25%. Calculations related to forecasting the Young’s modulus were performed with the use of the Mori-Tanaka model implemented in the Digimat MF software (analytical homogenization) and the ANSYS and Digimat FE software (numerical homogenization). Only for the filler content of 5%, an experimental verification was performed, where it was found that the Mori-Tanaka model is characterized by greater efficiency in predicting mechanical properties than the numerical method.

In the work of Pradhan et al. [56], the possibility of predicting the mechanical properties of composites with a polyester matrix filled with teak wood dust (TWD) powder was assessed. The mass content of fillers in the composite was as follows: 5, 10, 15, 20, and 25%. The obtained composites were tested for their mechanical properties. At the same time, the properties of the above-mentioned composites using the Digimat FE software were predicted. A high agreement of the results with the experiment was noted. A trend of slightly higher values of the simulation results compared to the experiment was noticed. The authors of the publication argued these results for the low content of voids in the actual structure of the composite.

In the work of Pradhan and Satapathy [57], micromechanical analyses were performed using the DIGMAT FE for a polyester resin—walnut shell powder (WSP) composite. The tested composites had the following filler contents: 4, 8, 12, 16, and 20%. The tensile strength was predicted and verified with the use of modeling, where slightly higher values were noted for the results of numerical analyses in relation to the results of experimental tests.

From the results of the analyses of Daramoul’s work [58], it is possible to analyze the influence of homogenization methods on the effectiveness of predicting the mechanical properties of a composite with an epoxy resin matrix filled with kaolinite microparticles of 6% mass fraction in the matrix. The computational methods were compared using the Digimat FE module, the Mori-Tanaka homogenization model, and the Voight model. For the 1st and 2nd method, the relative error between the simulation and the experiment for the tested Young’s modulus was 4.2%, and for Voight’s model, as much as 14.5%.

Isametova et al. [59] modeled the properties of a composite filled with short glass fibers with a mass content of 10, 20, and 30% in the polycarbonate matrix. It was noted that the average difference between the simulation and the experiment was approx. 7% in the context of the strength value for the stress-strain characteristic.

**Figure 12:**
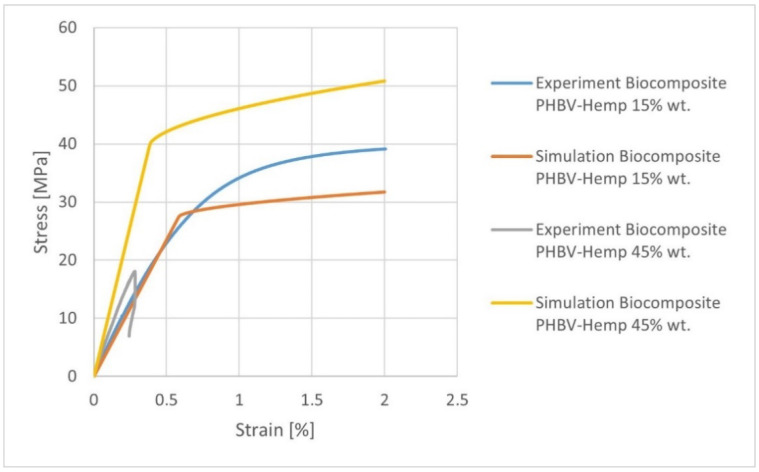
Stress-strain characteristics for various PHBV-hemp fiber biocomposites containing 15% (K15) and 45% (K45) mass filler.

When analyzing the discussed works, it was noted that the researchers predicted the properties of composites with a content of up to 30%. An interesting issue here pertains to level of the efficiency of the Mori-Tanaka model for the filler mass content of approx. 45%. Due to the above-mentioned results, the additional microstructural analyses related to the prediction of the properties of biocomposites for variable hemp fiber contents, i.e., 15 and 45 wt%, were performed. The 2nd order Mori-Tanaka homogenization model was selected for the calculations. The values of the fiber orientation tensor were adopted on the basis of numerical analysis for a biocomposite containing 30 wt% of filler. The results of the microstructural analyses are presented in Table 11. When analyzing the obtained results, it should be noted that the calculated value of Young’s modulus E1 for a biocomposite containing 15 wt% of the filler differs by approx. 10% from the experimental value ($\bar{x}$ = 5242.29 MPa, s = 196.29, and V = 3.74). On the other hand, for a biocomposite containing 45% of the filler, the Young’s modulus E1 differs by approx. 45% from the experimental value ($\bar{x}$ = 7162.28 MPa, s = 106.93, and V = 14.06). Taking into account the stress-strain characteristics (Figure 12), for a composite containing 15 wt% of the filler at a deformation of 2%, it was found that the stress values differ from the actual ones by about 23%. On the other hand, the obtained characteristic for biocomposites containing 45% of the filler completely differs from the course of the actual characteristic.

It can be seen that up to 30 wt% of fiber content, the results were very consistent with the experiment in the elastic range of stress-strain characteristic. This is confirmed in literature, where it was found that the Mori-Tanaka homogenization model was effective in microstructural calculations up to the filler content of about 25% by mass [29,30,31]. The obtained results and their degree of compliance (for 15 and 30% filler) prompted additional calculations related to the prediction of the properties of PHBV-hemp fiber biocomposites for the hemp fiber content: 5, 10, 20, and 25%, the results of which are presented in Table 12.

## 5. Conclusions

The properties of PHBV-hemp fiber biocomposites determined in the experimental studies allow for their use in the strength calculations of potential products, which are made of these biocomposites.

The use of the Mori-Tanaka analytical homogenization method allows for a good (up to a certain fiber content) prediction of the properties of PHBV-hemp fiber biocomposites. In the case of biocomposites filled with 15 wt% of fibers, a fairly good agreement of the calculation outcomes with the results of experimental tests was noted. This compliance would probably be greater if the actual values of the fiber orientation tensor from the numerical analysis of injection molding process were introduced.

For the biocomposite containing 45% of the filler, the prediction of their properties was not consistent with the experiment. It can be concluded that the calculation algorithm does not take into account the specific properties of cellulose fibers and composites filled with them, i.e., low wetting of the fibers with polymer when trying to introduce a higher fiber content (approx. 50%) and the fact that while increasing the fiber content, with further addition of cellulose filler, the porosity of the sample is increased and, to a lesser extent, the target volumetric fraction.

Moreover, the non-compliance of the results with the experiment for a biocomposite containing 45% of fibers may result from the assumptions that the Mori-Tanaka homogenization model is highly effective in predicting the properties of two-phase biocomposites with filler amounts up to 25%, and even higher values, where the threshold content has not been defined.

## Figures and Tables

**Table 1:** The Cross-WLF parameters of the K30 biocomposite model.

Parameter	Value
n_c_ [-]	0.122
τ* [Pa]	74657.9
D_1_ [Pa*s]	1.78 × 10^11^
D_2_ [K]	282.25
D_3_ [K/Pa]	0
A_1_ [-]	19.87
A_2_ [K]	51.6

**Table 2:** The Tait parameters of the K30 biocomposite model.

Parameter	Value
b_5_ [K]	433.15
b_6_ [K/Pa]	2.7 × 10^−8^
b_1m_ [m^3^/kg]	0.0007904
b_2m_ [m^3^/kgK]	8 × 10^−7^
b_3m_ [Pa]	7.96 × 10^7^
b_4m_ [1/K]	0.00704
b_1s_ [m^3^/kg]	0.0007489
b_2s_ [m^3^/kgK]	3 × 10^−7^
b_3s_ [Pa]	1.08 × 10^8^
b_4s_ [1/K]	0.002813
b_7_ [m^3^/kg]	4.8 × 10^−5^
b_8_ [1/K]	0.1202
b_9_ [1/Pa]	9.29 × 10^−9^

**Table 3:** The injection molding processing parameters.

Parameter	Value
Mold temperature [°C]	85
Melt temperature [°C]	185
Cooling time [s]	25
Packing time [s]	25
Packing pressure [MPa]	30
Injection rate [cm^3^/s]	35

**Table 4:** PHBV properties used in the calculations.

Parameter	Value
Density [kg/m^3^]	1250
Young’s Modulus [MPa]	2634
Poisson’s ratio [-]	0.35
Proportionality limit [MPa]	19.52
K [-]	23.167
n [-]	0.4551

**Table 5:** Hemp fiber properties used in the calculations [53,54].

Parameter	Value
Mass content of fibers [%]	30
Density [kg/m^3^]	1490
Specific heat [J/kg°C]	1000
Thermal conductivity [W/m°C]	1
Young’s Modulus E1 [MPa]	44,520
Young’s Modulus E2 [MPa]	44,520
Poisson’s ratio v12 [-]	0.12
Poisson’s ratio v23 [-]	0.12
Shear modulus G12 [MPa]	19,875
Thermal expansion coefficient α_1_ [1/°C]	1 × 10^−5^
Thermal expansion coefficient α_2_ [1/°C]	1 × 10^−5^
L/d ratio [-]	9

**Table 6:** RVE and hemp fibers geometry used in the calculations.

Parameter	Value
Fiber length [mm]	0.991
Fiber diameter [mm]	0.113
L/d ratio [-]	9
Mass content of fibers [%]	30
Fiber volume content, calculated [-]	0.276753
RVE dimensions [mm]	2 × 1 × 1
Voxel FE amount in RVE	250,000
Orientation tensor values:	
a_11_	0.6931
a_22_	0.1939
a_33_	0.113

**Table 7:** Visualization of fiber distribution in RVE for a defined value of the orientation tensor: Before (left) and after discretization (right).

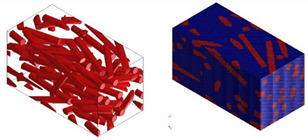	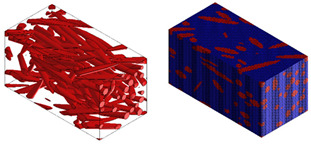
Fibers with cylindrical geometry	Fibers with ellipsoidal geometry
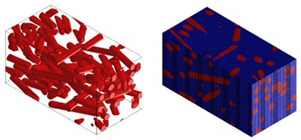	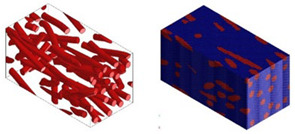
Fibers with sphero-cylindrical geometry	Fibers with twisted cylinder geometry

**Table 8:** Calculated values of the orientation tensor after converting data from Autodesk Moldflow Insight to RVE in Digimat software.

**Referenced values of orientation tensor (on the base of Autodesk Moldflow):**											
**a_11_**	**0.6931**	**a_12_**	**0**	**a_13_**	**0**						
		**a_22_**	**0.1939**	**a_23_**	**0**						
				**a_33_**	**0.113**						
											
Fibers with ellipsoid geometry:						Fibers with cylindrical geometry:					
a_11_	0.77	a_12_	0.073	a_13_	0.088	a_11_	0.77	a_12_	0.11	a_13_	0.031
		a_22_	0.16	a_23_	0.0061			a_22_	0.18	a_23_	0.018
				a_33_	0.074					a_33_	0.006
											
Fibers with sphero-cylindrical geometry:						Fibers with twisted cylinder geometry:					
a_11_	0.71	a_12_	−0.087	a_13_	0.005	a_11_	0.82	a_12_	−0.013	a_13_	0.052
		a_22_	0.19	a_23_	−0.0069			a_22_	0.11	a_23_	−0.0053
				a_33_	0.088					a_33_	0.07
											

**Table 9:** The predicted properties of the biocomposite PHBV-hemp fiber for variable types fiber geometry in RVE.

Properties	Fiber Geometry Model in RVE			
	Sphero-Cylindrical	Cylindrical	Elipsoid	Twisted Cylinder
Density [kg/m^3^]	1280	1280	1280	1270
Young’s Modulus E1 [MPa]	5699.9	5704.1	6561.2	5480.1
Young’s Modulus E2 [MPa]	3943.2	3816.3	4147.2	3599.5
Young’s Modulus E3 [MPa]	3868.7	3785.4	4164.6	3563.6
Poisson’s ratio v12	0.347	0.371	0.384	0.339
Poisson’s ratio v21	0.240	0.248	0.243	0.223
Poisson’s ratio v13	0.324	0.303	0.285	0.332
Poisson’s ratio v31	0.219	0.201	0.181	0.216
Poisson’s ratio v23	0.379	0.376	0.367	0.398
Poisson’s ratio v32	0.372	0.373	0.360	0.394
Kirchhoff’s module G12 [MPa]	1635.5	1620.3	1837.7	1421.1
Kirchhoff’s module G23 [MPa]	1489.2	1322.3	1415.5	1367.5
Kirchhoff’s module G13 [MPa]	1378.7	1248.9	1306.2	1277.7

**Table 10:** The predicted mechanical properties of the biocomposite PHBV-hemp fiber (K30) for a variable homogenization method.

Properties	Homogenization Model			
	Mori-Tanaka (1st Order)	Mori-Tanaka (2nd Order)	Double Inclusion	Numerical Homoge-Nization
Density [kg/m^3^]	1290	1290	1291	1280
Young’s Modulus E1 [MPa]	7232.7	7232.7	7625.2	6561.2
Young’s Modulus E2 [MPa]	4760.7	4760.7	4996.6	4147.2
Young’s Modulus E3 [MPa]	4571.4	4571.4	4793.7	4164.6
Poisson’s ratio v12	0.329	0.329	0.323	0.384
Poisson’s ratio v21	0.217	0.217	0.212	0.243
Poisson’s ratio v13	0.323	0.323	0.318	0.285
Poisson’s ratio v31	0.204	0.204	0.199	0.181
Poisson’s ratio v23	0.380	0.380	0.382	0.367
Poisson’s ratio v32	0.365	0.365	0.366	0.360
Kirchhoff’s module G12 [MPa]	2016.8	2016.8	2136.8	1837.7
Kirchhoff’s module G23 [MPa]	1863.8	1863.8	1973.6	1415.5
Kirchhoff’s module G13 [MPa]	1692.8	1692.8	1773.8	1306.2

**Table 11:** The predicted properties of the biocomposite PHBV-hemp fiber for variable types of fiber geometry in RVE.

Properties	Mass Content of Hemp Fibers [%]		
	15	30	45
Density [kg/m^3^]	1270	1290	1310
Young’s Modulus E1 [MPa]	4711.3	7232.7	10,380
Young’s Modulus E2 [MPa]	3576.5	4760.7	6377
Young’s Modulus E3 [MPa]	3495	4571.4	6057.4
Poisson’s ratio v12	0.342	0.329	0.313
Poisson’s ratio v21	0.259	0.217	0.192
Poisson’s ratio v13	0.337	0.323	0.304
Poisson’s ratio v31	0.250	0.204	0.177
Poisson’s ratio v23	0.379	0.380	0.369
Poisson’s ratio v32	0.370	0.3651	0.351
Kirchhoff’s module G12 [MPa]	1431.4	2016.8	2806.7
Kirchhoff’s module G23 [MPa]	1360.2	1863.8	2559.7
Kirchhoff’s module G13 [MPa]	1282.7	1692.8	2273.8

**Table 12:** The mechanical properties prediction of the biocomposite PHBV-hemp fiber for the mass share of fibers: 5, 10, 20, and 25%.

Properties	Mass Content of Hemp Fibers [%]			
	5	10	20	25
Density [kg/m^3^]	1256.7	1263.5	1277.4	1284.4
Young’s Modulus E1 [MPa]	3285.1	3976.1	5495.3	6333.6
Young’s Modulus E2 [MPa]	2930.8	3243.0	3936.7	4329.3
Young’s Modulus E3 [MPa]	2907.1	3192.0	3821.9	4178.5
Poisson’s ratio v12	0.34751	0.3447	0.33804	0.33413
Poisson’s ratio v21	0.31003	0.28114	0.24216	0.22839
Poisson’s ratio v13	0.34602	0.34185	0.33282	0.32789
Poisson’s ratio v31	0.3062	0.27443	0.23147	0.21632
Poisson’s ratio v23	0.36519	0.37409	0.38119	0.38145
Poisson’s ratio v32	0.36224	0.3682	0.37008	0.36817
Kirchhoff’s module G12 [MPa]	1116.1	1267.5	1609.4	1803.6
Kirchhoff’s module G23 [MPa]	1093.4	1221.2	1512.2	1679.2
Kirchhoff’s module G13 [MPa]	1069.1	1171.1	1405.6	1541.5

## Data Availability

The data presented in this study are available on request from the corresponding author.
